# Advanced Genomics-Based Approaches for Defining Allograft Rejection With Single Cell Resolution

**DOI:** 10.3389/fimmu.2021.750754

**Published:** 2021-10-14

**Authors:** Tiffany Shi, Krishna Roskin, Brian M. Baker, E. Steve Woodle, David Hildeman

**Affiliations:** ^1^ Division of Immunobiology, Cincinnati Children’s Hospital Medical Center, Cincinnati, OH, United States; ^2^ Department of Pediatrics, University of Cincinnati College of Medicine, Cincinnati, OH, United States; ^3^ Immunology Graduate Program, University of Cincinnati College of Medicine, Cincinnati, OH, United States; ^4^ Medical Scientist Training Program, University of Cincinnati College of Medicine, Cincinnati, OH, United States; ^5^ Division of Biomedical Informatics, Cincinnati Children’s Hospital Medical Center, Cincinnati, OH, United States; ^6^ Department of Chemistry and Biochemistry and the Harper Cancer Research Institute, University of Notre Dame, Notre Dame, IN, United States; ^7^ Division of Transplantation, Department of Surgery, University of Cincinnati College of Medicine, Cincinnati, OH, United States

**Keywords:** transplantation, allograft rejection, genomics, sequencing, single cell RNA seq

## Abstract

Solid organ transplant recipients require long-term immunosuppression for prevention of rejection. Calcineurin inhibitor (CNI)-based immunosuppressive regimens have remained the primary means for immunosuppression for four decades now, yet little is known about their effects on graft resident and infiltrating immune cell populations. Similarly, the understanding of rejection biology under specific types of immunosuppression remains to be defined. Furthermore, development of innovative, rationally designed targeted therapeutics for mitigating or preventing rejection requires a fundamental understanding of the immunobiology that underlies the rejection process. The established use of microarray technologies in transplantation has provided great insight into gene transcripts associated with allograft rejection but does not characterize rejection on a single cell level. Therefore, the development of novel genomics tools, such as single cell sequencing techniques, combined with powerful bioinformatics approaches, has enabled characterization of immune processes at the single cell level. This can provide profound insights into the rejection process, including identification of resident and infiltrating cell transcriptomes, cell-cell interactions, and T cell receptor α/β repertoires. In this review, we discuss genomic analysis techniques, including microarray, bulk RNAseq (bulkSeq), single-cell RNAseq (scRNAseq), and spatial transcriptomic (ST) techniques, including considerations of their benefits and limitations. Further, other techniques, such as chromatin analysis *via* assay for transposase-accessible chromatin sequencing (ATACseq), bioinformatic regulatory network analyses, and protein-based approaches are also examined. Application of these tools will play a crucial role in redefining transplant rejection with single cell resolution and likely aid in the development of future immunomodulatory therapies in solid organ transplantation.

## Introduction

There are over 150,000 organs transplanted annually worldwide (1), with numbers increasing yearly. The optimal solution for organ failure is transplantation, however, organ transplantation requires lifelong immunosuppressive therapy. In the absence of adequate immunosuppression, acute rejection may develop, which may be driven by T lymphocytes [acute cellular rejection (ACR)], by antibody [antibody mediated rejection (AMR)], or by both T lymphocytes and antibody [mixed acute rejection (MAR)]. In addition to acute rejection, chronic rejection may also develop, which involves not only inflammatory cell infiltration into the graft, but also vasculopathy from repeated injury and repair (2). While standard immunosuppression (IS) is effective at decreasing CD8+ T cell driven ACR, excess IS may lead to malignancies and infectious complications (3). Additionally, current standard-of-care immunosuppressive therapies, including calcineurin inhibitors (CNIs), corticosteroids (CCS), and mycophenolate mofetil (MMF), have a multitude of toxicities (4), including nephrotoxicity, neurotoxicity, gastrointestinal toxicity, as well as metabolic derangements. These adverse effects reduce quality of life and may contribute to decreased patient and allograft survival (5, 6). Intriguingly, although CNI/CCS/MMF-based regimens have been used as the primary means for maintenance immunosuppression for decades now, molecular insights governing graft acceptance and rejection remain incompletely defined, especially for individual graft resident and infiltrating immune cell populations.

Given the role of T cells in ACR, novel biologic therapies focusing on T cell co-stimulatory blockade, such as belatacept (CTLA4-Ig) (7) or iscalimab (anti-CD40 mAb) (8) have been developed in hopes of decreasing treatment-mediated toxicities while maintaining tolerance to the allograft. Notably, for the first time under any maintenance immunosuppressive regimen, long-term kidney transplant function improved under belatacept as compared to the time of transplant (9). This shows the promise of belatacept in reducing the nephrotoxicity that can directly lead to decreased kidney allograft function. However, there is an increased rate of rejection under these new biologics. Under standard CNI and MMF combination therapy, 1-year rejection rates are roughly 10-15% (10, 11), while rejection rates under belatacept are roughly 20-25% (9, 12), when accompanied with anti-thymocyte globulin, which targets T cells, or alemtuzumab, an anti-CD52 monoclonal antibody against lymphocytes, as induction therapy (12). In addition to increased rejection rates, rejection under belatacept IS is more difficult to treat with conventional anti-rejection therapies, such as high dose corticosteroid therapy and anti-lymphocyte globulin therapy (13). Therefore, there is still a need for a therapy that can both limit treatment-mediated toxicities and decrease rejection rates.

Rejection episodes can also be challenging to classify, with rejection classification and grading relying on visualization of infiltrate and tissue damage *via* histological sectioning and staining, subjecting it to considerable inter-observer bias (14, 15). A particular issue is that of “sampling bias”, which accounts for the fact that rejection can exist as focal regions and not be uniform throughout the allograft. Thus, depending on the location of the biopsy, rejection may not be visualized even though it is occurring in another region of the allograft. Further, rejection markers, such as serum creatinine level and white cell count, typically rely on peripheral blood sampling, which may not accurately represent histological severity nor reflect what is happening in the rejecting allograft. Additionally, renal allograft dysfunction may occur as a result of processes other than rejection, such as dehydration or infection. This prompts the need to understand the underlying mechanisms employed by cells within the rejecting organ.

Recent development of novel genomics tools combined with powerful bioinformatics approaches has enabled the characterization of inflammatory processes that underlie transplant rejection. These approaches are beginning to provide profound insights into rejection biology, including individual residential and infiltrating cellular transcriptomes and cell-cell interactions. Here, we will discuss the available transcriptomic methods as well as considerations of benefits and limitations of these techniques (**Table 1**). Application of these techniques will play a crucial role in redefining transplant rejection and likely aid in the development of innovative and targeted immunomodulatory therapies for mitigating and preventing solid organ transplant rejection.

**Table 1:** Advantages and Disadvantages of Advanced Geomics-Based Approaches.

Technology	Advantages	Disadvantages	References
Microarrays	• widespread use, leading to many validation datasets • cost-effective compared to other techniques • simpler, more streamlined analysis • can be performed using FFPE tissue • identified ARTs available	• analyses are supervised • cannot determine which cells particular transcripts are from • no combined protein analyses • no TCR/BCR analyses • lacks histological context	(16, 17)
BulkSeq	• more quantitative analyses compared to microarrays • does not rely on probe hybridization • can be performed using FFPE tissue • better detection of lowly expressed transcripts • allows for identification of SNPs	• cannot determine which cells particular transcripts are from • limited analysis of complex transcriptomes • cannot determine cell specific TCR/BCR sequences • rejection-driving transcripts may be drowned out • lacks histological context	(18–20)
scRNAseq	• provides transcriptomic information of single cells • can identify novel cell-type specific transcripts • allows for identification of SNPs • provides insight into the clonal immune response during rejection • can perform paired TCR/BCR analysis • outlines pathway toward alloantigen identification • many software programs available for in-depth intercellular communication and cellular differentiation analyses	• difficult tissue preservation • difficult tissue digestion and single cell isoaltion • lacks histological context	(21–27)
Methods coupled to scRNAseq scATACSeq	• insight into gene regulatory networks • allows for cell lineage analyses	• open chromatin sites captured may be limited • high background noise	(28–33)
mass cytometry/cyTOF	• provides insight into protein expression		
CITEseq/REAPseq	• combined mRNA & protein expression in of cells	• supervised protein analyses via antibody selection	
Index Sorting	• combined mRNA & protein expression in of cells	• lower throughput limited by flow rate	
Spatial Transcriptomics	• interrogates cellular interactions in their native context • no tissue dissociation requirements • allows determination of Banff criteria on same tissue slice as transcriptomic data • can be performed using FFPE tissue • hybridization-based approaches allow for single cell resolution • can incorporate TCR/BCR profiling • combination with CODEX or Imaging Mass Cytometry allows for protein expression in tandem	• hybridization-based approaches requires a priori knowledge of genes of interest • RNA from FFPE tissue may be chemically modified or degraded	(34–38)

## Targeted Transcriptomics

The development of microarray technology, at the time, was a revolutionary breakthrough because it allowed simultaneous assessment of expression of thousands of genes. Microarray analyses provide assessment of gene expression from cDNA libraries generated after mRNA isolation from tissue or from purified cells. The cDNA is fluorescently labeled, with differing labels if desired to distinguish individual samples, before hybridization on a chip that has been previously loaded with a large array of oligonucleotide probes, each specific for an individual gene. This method (16) helps create a gene expression profile of the samples and allows for parallel detection of specific genes in a single reading, which provides insight into the differential gene expression between individual samples. Additionally, this standardized protocol (16), along with the ease and speed of analysis, made microarrays a useful tool in transplant research.

The use of microarrays in transplantation has enabled the identification of transcripts that correlate with rejection, allowing gene expression data to be interpreted in conjunction with histologic data to grade rejection. Microarray techniques have been leveraged in multiple ways to not only examine the profile of transplant rejection (39–41), but also identify serum protein biomarkers, such as PECAM1, that correlate with acute rejection (42) and autoantibodies that may play a role in rejection (43). Indeed, many studies utilizing microarray techniques have been done to study AMR (44–46). Microarray techniques were also one of the first techniques used to identify C4d-negative AMR in kidney transplantation through correlation of increased expression of renal endothelial transcripts in the allograft with biopsy proven acute rejection (47). Microarray analyses of AMR in the INTERCOM study revealed that that results of a microarray-based test using kidney biopsies correlated strongly with conventional assessment methods of AMR and could also predict early progression to transplant failure (45).

In addition to AMR, microarrays have been used to characterize T cell mediated rejection (TCMR). In particular, identification of cytotoxic T cell transcripts involved in rejection may aid in the estimating T cell infiltration in the rejecting allograft (48). TCMR-related transcripts show increased expression of genes related to effector T cells and co-stimulation, as well as macrophage activation, providing insight into potential cellular pathways involved in rejection. Further, TCMR related transcripts are highly ranked in relation to transcripts associated with rejection universally but low in AMR related transcripts, suggesting that microarray transcripts seen in rejection are likely to come from TCMR related genes. Therefore, when analyzing rejection as a whole, TCMR related transcripts may be overrepresented. However, it is still unclear proportionally how much this gene expression contributes to the overall transcriptomic profile (14, 49, 50).

The widespread use of microarray techniques in transplant had led to the development of the Molecular Microscope™ to describe transplant rejection. Through comparison of transcripts from rejecting allografts versus non-rejecting organs, acute rejection-associated transcripts (ARTs) have been identified (51). This data has identified genes whose expression correlates with rejection, which helps infer the cell populations involved. Identification of ARTs also help distinguish between transcripts that are truly involved in rejection and those that are increased due to organ or vascular injury, which can occur without rejection. This has enabled the identification of universal rejection transcripts, which are largely comprised of interferon gamma (IFNγ)-inducible genes (52, 53). Importantly, ARTs have been shown to be conserved between kidney and heart allograft transplant rejection (44, 54), suggesting that ARTs may broadly represent common mechanisms for solid organ allograft rejection. Furthermore, almost all ARTs described are also found in the universal rejection transcripts (53), confirming the role of these IFNγ-inducible genes in acute transplant rejection. In addition to the identification of ARTs, integration of transcriptional data from different organ rejections have also enabled the identification of a common rejection module (CRM), comprised of chemokines that control immune cell trafficking (55). In this study, CRM expression was shown to correlate not only with serum creatinine levels from rejecting kidneys, but also the extent of allograft injury in acute rejection. By combining multiple datasets to identify ARTs and CRMs, microarray technology can be used in combination with histological Banff grading of rejection to create a more sensitive and specific way to monitor allograft acceptance and rejection.

In addition to furthering the understanding of transplant rejection, microarrays also have the potential to be an important clinical monitoring tool for transplant patients by providing a large amount of data with a relatively fast turnaround time (56, 57). These studies suggest that microarray analyses provide useful information regarding increased expression of genes in transplant rejection. A key component in these studies is the choice of the control sample for evaluating studies of transplant rejection genomics. Initial studies using microarray techniques in kidney transplant rejection compared the transcriptomic profiles of rejectors versus non-rejectors, but these non-rejector controls were from un-transplanted nephrectomies or kidneys with carcinomas (51, 58). Since the control samples are taken from un-transplanted kidneys, the gene expression readout could be drastically skewed, as the kidney was never placed in an allogeneic recipient. Indeed, this approach does not take into consideration genetic signatures of injury from the transplant surgery (e.g., ischemia/reperfusion, etc.) or the presence of recipient immune cells in the biopsy. This issue was addressed in subsequent studies that focused on using biopsies taken from patients that underwent a transplant but no rejection as controls (44, 45, 59, 60), which focused differences observed to rejection rather than transplantation-related factors.

However, there are still substantial limitations with the use of microarrays (**Table 1**). With Affymetrix technology, the whole genome can be interrogated, but NanoString offers a cost-effective way to interrogate genes of interest. In contrast to microarray techniques, NanoString does not require cDNA synthesis and amplification, as it directly assesses mRNA through hybridization thereby avoiding potential amplification bias (17). However, this limits discovery as genes investigated are pre-determined by the investigator. Further, due to the aggregate nature of the analyses, it is not possible to determine which cells particular transcripts came from, limiting the ability to determine cellular drivers and mechanistic cell-cell interactions and intracellular pathways of rejection. For example, due to shared similarities in inflammatory genes between TCMR and AMR, microarray analyses reveal an inability to reliably distinguish between these two drivers of rejection (61, 62). Furthermore, both ACR and AMR can coexist as MAR in biopsy samples (63). Additionally, despite the identification of ARTs, many signals associated with rejection were also associated with injury-induced biopsies without rejection (62). Therefore, these results outline a need for additional approaches that allow unbiased transcript analysis and linkage of transcripts to their cellular source.

## Bulk RNA Sequencing

One method that would help overcome the limitation of supervised analyses of gene sets with microarrays is the use of bulk RNA sequencing (bulkSeq). BulkSeq involves generation of a cDNA library from a mixed sample of cells or whole tissue, and high-throughput sequencing. Typically, cells or tissues are first stored in an RNA-stabilizing solution to protect the RNA integrity before RNA isolation. However, more recent technological developments have enabled RNA isolation from formalin-fixed, paraffin-embedded (FFPE) histological tissue blocks (18). This has enabled the ability to perform RNA analyses using the aforementioned microarray techniques or bulkSeq. Despite this advancement, which opens the door for in-depth, retrospective analyses of preserved samples, formalin fixation of the tissue may lead to inferior RNA quality (64, 65), producing artificial associations and overrepresentation of histone transcripts (66).

In contrast to microarrays, bulkSeq does not require transcript-specific probes, allowing for a more quantitative analysis that does not rely on hybridization. Moreover, bulkSeq quantifies all transcripts in a given tissue or cell population, which has the advantage of identifying potentially novel transcripts that may not have been thought to contribute to disease (67). This results in a more unbiased approach to understanding the gene expression of a sample. Due to these benefits over microarray techniques, there has been a movement toward these types of high-throughput sequencing approaches.

Initial studies directly compared microarray technology to bulkSeq technology to determine the sensitivity and specificity of the two different methods. These studies showed comparable similarities between bulkSeq and microarray analyses (19) and also revealed a strong correlation in the data obtained using both techniques in peripheral blood and tissue compartments (68). These comparison studies confirm that bulkSeq techniques are able to capture data comparable to those obtained from microarrays while also overcoming the limitations set by microarrays. Two independent studies found that bulkSeq allows for more sensitive and specific detection of transcripts, which provides for a more quantitative analysis of differential gene expression and also detection of genes with lower expression levels (69, 70). Indeed, with microarrays, lowly expressed genes may be masked by the background interference, while highly expressed genes may not be fully quantitative due to probe hybridization saturation (71, 72). BulkSeq techniques overcome these limitations through better detection of lowly expressed transcripts (73) and the depth of sequencing allows for a broader range of quantitation of gene expression.

BulkSeq approaches have been instrumental in better characterizing the gene expression profiles associated with organ rejection (74, 75) by incorporating the ability to identify donor versus recipient transcripts based on single nucleotide polymorphisms (SNPs) (76) – something that cannot be detected using microarrays. It is important to note that SNPs can also affect microarray performance, as a majority of studies have been performed in people of a white, European background. Using bulkSeq, one group found that the ratio of heterozygous to homozygous variants from a non-reference genome and mRNA expression can be used to estimate and identify cellular infiltration (76), which strongly correlates with rejection. These data showed the capability of bulkSeq to identify variants that help further classify allograft infiltration. Identification of SNPs is a hallmark feature of bulkSeq that microarray technologies are incapable of, providing greater insight into the genetic interplay involved in rejection.

In addition to identifying nucleotide variants that may play a role in rejection, bulkSeq techniques have also enabled identification of new targets and determination of cellular response to potential immunosuppressive therapies. One study used bulkSeq to identify the effects of various anti-CD3 antibodies on the transcriptomes of T cells *in vitro*, showing changes in genes associated with cellular proliferation, DNA metabolic processes, and cytokine secretion after treatment (77). Deeper analyses revealed differentially expressed genes and immune-associated pathways between the different antibody therapies, indicating how bulkSeq can be used to assess the effects of similar immunosuppressive therapies on cell populations. This can provide potential indications of optimal therapies for individual patients. Thus, the use of bulkSeq has enabled an unbiased assessment of gene expression changes occurring after immunosuppressive therapy, identifying critical pathways targeted as well as off-target effects. This can drastically improve the current understanding of how anti-rejection medication affects the cellular mechanisms responsible for rejection.

In addition to whole transcriptomic sequencing, bulkSeq techniques have also been applied to determine the B lymphocyte or T lymphocyte repertoire. T cells recognize peptides in the context of human leukocyte antigens (HLA), whereas B cells recognize native protein structures. Each T cell receptor (TCR) has two chains, an alpha chain and a beta chain, and each B cell receptor (BCR) also has two chains, a heavy chain and a light chain. To get the true identity of an individual clone, both chains of a TCR or BCR must be sequenced, since an individual beta or heavy chain can pair with more than one alpha or light chain. Additionally, sequencing only one of the chains of a TCR or BCR will only enable identification of the number of transcripts and only gives an estimation on the frequency of individual clones. Initial attempts at BCR repertoire identification, termed Ig-seq, included bulk DNA or RNA sequencing of the immunoglobulin heavy chain and light chain, but cellular clonal frequencies were difficult to determine with these bulk approaches (20). Further, the heavy and light chain pairings were lost using this approach, limiting the ability to identify true clonality. Nevertheless, the Ig-seq approach has been adapted to characterize the B cell repertoire in transplantation. BCR Sequencing (BCRseq) has been used to characterize the B cell repertoire in kidney (78) and heart (79) transplant, which has enabled identification of common “ancestor” molecules of B cell clones. Despite only immunoglobulin heavy chain sequencing, these studies provided preliminary insight into clonal expansion in transplantation and its relationship to clones identified. Similarly, high throughput sequencing of the TCR beta chain (80) has been done to identify the T cell repertoire in combined kidney and bone marrow transplantation (81) and liver transplantation (82). However, due to the transcripts coming from a mixed population of cells obtained from bulkSeq and only single chain sequencing, specific T cell receptor (TCR) or B cell receptor (BCR) clones that are responsible for TCMR or AMR cannot be identified. Therefore, true assessment of individual clonal expansion and deletion cannot be assessed with these methods.

Despite the benefits of bulkSeq, it shares a limitation with microarray technology: the ability to determine gene expression on the individual cell level (**Table 1**). This limitation prevents the ability to determine the quality and quantity of immune (and non-immune) cells within the rejecting allograft. Additional challenges of bulkSeq include shorter read mapping, leading to limited analysis of the transcriptomes of more complex samples (71). Investigating the cell-specific gene expression is crucial to understanding the mechanisms of rejection and providing insight into potential pathways to be targeted to mitigate rejection episodes. Furthermore, the cells driving rejection are very small in number, and may be drowned out in bulkSeq approaches. Therefore, the movement toward single-cell RNA sequencing has opened a new era in the field of transplant genomics.

## Single Cell RNA Sequencing

While bulkSeq and microarray approaches can identify gene expression within whole tissues, single cell RNA sequencing (scRNAseq) approaches give unprecedented clarity to gene expression patterns in tissues. With scRNAseq, tissue preservation plays an integral role in the ability to isolate quality RNA from the tissue. For biopsy samples, we and others have shown that the tissue can be either processed fresh or preserved in a solution, typically a DMSO-based medium, that allows for the sample to be frozen down in an insulated container with an isopropanol chamber to facilitate slow freezing (21, 83). This approach allows the sample to then be thawed and processed at a later time which is advantageous for multi-site clinical trials. Preliminary experiments to perform scRNAseq using RNA extracted from FFPE tissue have also been attempted (84), but given the challenges of using FFPE tissue in microarray and bulkSeq analyses, further optimization is necessary.

In contrast to bulkSeq and microarrays, scRNAseq presently requires generation of a single-cell suspension from the sample in question. This presents a large challenge to analyze allograft tissue, as tissue dissociation protocols typically require digestion with enzymes that are active at 37˚C, resulting in potential heat-induced artifacts. Recently, a novel, cold-active protease digestion protocol (22) has been developed to mitigate these effects, and has allowed the generation of high-quality scRNAseq data. Following tissue dissociation, either plate-based (23) limiting dilution or microfluidics (85) approaches are used for droplet formation following generation of a single-cell suspension. In each droplet, a cell is associated with oligoDTs to capture RNA from the individual cells, along with bar codes unique to each droplet. After cellular lysis and labeling of transcripts with unique molecular identifiers (UMIs), the captured RNA is reverse transcribed and amplified, generating cDNA libraries with each cell possessing its own unique UMI. Then, the cDNA can be amplified and sequenced. In contrast, methods that do not require droplet formation or UMIs can also be performed. For example, fluorescence-activated cell sorting (FACS) can also be utilized to sort cells into individual wells of a 96- or 384-well plate, with PCR and barcoding occurring in the individual wells of the plate (80, 86). Following sequencing, the data can be analyzed and visualized using several bioinformatic-based software programs.

Multiple droplet-based microfluidics approaches have been used, including DropSeq (87) and InDrops (88, 89). These techniques involve capturing individual cells and labeling their mRNA with a unique barcode following cell lysis to track the cellular origin of transcripts. DropSeq involves creation of a droplet that consists of the cell and barcoded microparticle beads, while InDrops uses a hydrogel droplet consisting of barcoded primers that combine with cells in droplets. Both of these techniques enable the parallel analysis of thousands of genes in thousands of individual cells from a heterogeneous population. However, an advantage of InDrops over DropSeq is that InDrops is able to barcode a much higher fraction of cells that are run through the system (89). Notably, both DropSeq and InDrops can be done in the laboratory using widely available equipment, making these a more affordable approach to single cell transcriptomics, relative to the newer, more streamlined approaches. Fluidigm C1 single cell mRNA sequencing technology utilized techniques learned from DropSeq and InDrops while also allowing for TCR determination *via* TraCeR analysis pipeline following full-length mRNA sequencing (90). 10X Genomics (24) offers a similar approach to Fluidigm C1 and also offers TCR and BCR sequencing using their 5-prime V(D)J kit. However, there has been a movement toward 10X Genomics’ pipeline due to their platform’s ability to assess thousands of cells, versus only hundreds of cells using Fluidigm-based techniques.

The ability to analyze the transcriptomic profile of single cells has already proved to be groundbreaking in the characterization of tissues in other disease processes. scRNAseq of tumors has uncovered a multitude of specific cell populations in serous epithelial ovarian cancer (91), with unique cell populations present depending on the grade of the tumor and whether or not the tumor was metastatic. Similar experiments were conducted to identify gene expression signatures of tumor epithelial and endothelial cells in breast cancer (92), glioblastoma (93), renal carcinomas (94) and head and neck cancer (95). Furthermore, scRNAseq has also been leveraged to describe the tumor-infiltrating myeloid populations across different tumor types (96). By identifying the similarities and differences between common cancer types, this approach has increased our fundamental understanding of the role played by myeloid cells in tumor progression. In addition to furthering the understanding of tumor biology, scRNAseq techniques have been implemented to better understand chronic, progressive diseases such as lupus nephritis (21) or focal segmental glomerulonephritis (FSGS) (97). Similar to the studies done with tumors, these studies identified a multitude of immune cell populations, and were even able to capture intermediate states of activation (21). Furthermore, identification of novel cell-type specific transcripts (97) has greatly contributed to the single cell atlas of organs. Due to the successes in other fields, there is great potential for the use of scRNAseq for breakthroughs in solid organ transplantation.

Preliminary studies using scRNAseq to understand organ rejection have already provided valuable insight into the pathology, including the use of this technique to understand the lymphocytic infiltration involved in AMR after renal transplantation (98, 99). Further, the use of scRNAseq to map out the transcriptomic profile of pulmonary fibrosis of lung explants prior to transplantation (100) proved to be a valuable first step to providing comparators for future studies involving the fibrosis of chronic lung rejection. scRNAseq analysis of lung transplantation has revealed the presence donor-derived T cells with a tissue-resident memory phenotype in the bronchoalveolar lavage fluid of non-rejecting patients, which suggests that donor T cells are present in the lung allograft even in the absence of rejection (101). Further, the authors found that slower replacement these donor T cells by recipient T cells correlated with a lower incidence of rejection. Importantly, this study did not find persistence of donor-derived memory T cells in the peripheral blood, indicating resident memory T cell turnover. Altogether, these conclusions suggest that the balance between donor and recipient T cells is important for allograft tolerance. Another study also looked at the role of donor-derived T cells, but in the context of intestinal transplantation. Here, the authors assessed how donor-derived tissue resident T cells can help achieve allograft tolerance by destruction of recipient hematopoietic cells (102). Since tissues such as the lung and intestine may have more resident memory T cells, graft-versus-host (GvH) disease is more commonly seen following lung and intestinal transplantation as compared to other solid organ transplantation. However, this study utilizes scRNAseq techniques, combined with TCR sequencing, to show that GvH may not necessarily be a negative complication. Intriguingly, the authors showed that graft-derived GvH-reactive donor T cells migrate into recipient circulation and bone marrow, promoting engraftment of graft-derived stem cells to maintain mixed chimerism and promote immune tolerance. These two studies indicate there is great potential in understanding donor-derived T cells, as they may play an integral role in allograft tolerance.

In AMR and TCMR, B cells and T cells play a crucial role in the rejection process, so sequencing the B cell receptor (BCR) or T cell receptor (TCR) in the context of scRNAseq studies would provide information about the clonality and the paired chain CDR3 sequences that determine receptor specificity, potentially leading to the identification of targets that are recognized. Notably, targets in TCMR remain poorly characterized and poorly understood. For decades, alloreactive T cells driving direct ACR have been viewed as either responding primarily to unique determinants on allo-HLA (HLA-centric responses), or to a plethora of non-self peptides presented by allo-HLA (peptide-centric). Recent data however has suggested that many alloreactive T cells are allospecific, responding to unique peptide-HLA complexes present on the surface of allografts and displaying similar levels of specificity towards their targets as do conventional T cells (103). Therefore, alloreactive T cells may be reactive toward allo-peptides and viral peptides, while allospecificity refers to reactivity only toward allo-peptides. Indeed, structural and biochemical studies have shown that the binding of alloreactive TCRs can be dependent upon unique features of both the allo-peptide and the allo-HLA, mimicking how TCRs recognize pathogen-derived peptides presented by self-HLA (104). While there is increasing clarity around the functional, biochemical, and structural basis of TCR recognition of allo-peptide/HLA in general, the lack of knowledge regarding the specificities of alloreactive T cells is a major reason for our limited biologic insights into TCMR.

Importantly, the ability to determine CDR3 sequences from T cell clones driving TMCR may facilitate identification of allo-peptides with the assistance of novel informatics or screening tools, such as GLIPH (25) or combinatorial peptide libraries. GLIPH uses an algorithm to identify shared motifs within CDR3 sequences, which helps to organize these sequences and cluster TCRs based on CDR3 similarity (25). This could eventually enable the prediction of TCR binding to MHC-restricted peptide. Further, screening with combinatorial peptide libraries could enable the identification of the peptide antigen that a particular TCR recognizes. A major breakthrough in T cell biology was the identification of T cell epitopes, enabling MHC tetramers. MHC tetramers are complexes of four identical MHC-peptide pairs bound to a fluorochrome, enabling the binding of 3-4 TCRs at once, allowing increased avidity and staining intensity (105). This development has allowed for the tracking and analysis of T cells from activation to memory development in infection (106, 107), cancer (108), and autoimmunity (109–111). Identification of allo-peptides may facilitate similarly groundbreaking studies of T cells driving direct ACR.

Utilization of scRNAseq not only enables the transcriptomic description of these cells responsible for rejection, but also provides the opportunity to study the allospecificity and track T and B cell clonal dynamics. Studies of cardiac cellular rejection have shown that graft infiltrating T cells overlapped with those found in the peripheral blood, but interestingly, there was minimal overlap of the B cells between the two compartments (112). The authors used this data to suggest that there is B cell clonal expansion in the allograft, which could occur as part of a well-known phenomenon of germinal center-like structures present in chronically rejecting allografts (113, 114). However, it is unclear if these GC-like structures are sufficient to promote class-switch recombination or somatic hypermutation. Other studies have also begun to identify and track alloreactive TCRs in intestinal transplantation by using recipient blood responding to donor-derived lymphocytes in a mixed lymphocyte reaction (MLR) (115). Dividing donor-reactive cell proliferation can be tracked with fluorescent dyes and proliferated cells can then be sequenced and “fingerprint” donor-reactive TCRs.

In addition to using the sequences to identify potential alloreactive, graft-infiltrating T cells, sequencing of TCRs can also help identify virus-specific T cells and their associated HLA alleles (116). Differentiating allo- versus virus-specific T cells will help distinguish between clonally expanded intragraft T cell populations and enable the characterization of the transcriptomic profile of truly alloreactive clones. Also complicating the interpretation of specificity is the fact that many alloreactive T cells can also have viral-specificity due to TCR degeneracy (117, 118). However, a limitation of these studies is that they only have the TCR beta chain and B cell immunoglobulin heavy chain sequenced, limiting the ability to truly track clonality, and in the case of T cells, limits antigen discovery and determination of true alloreactivity. To help with this, one group has developed and used VDJPuzzle to reconstruct the TCR alpha and beta chain from scRNAseq data to help identify hepatitis C-specific T cells (119). The challenge of using such an approach in transplantation and identifying alloreactive T cells is that the antigens and epitopes are still unknown. Determination of proper TCR alpha and beta chain pairs enables the determination of target peptides, either allo- or viral-reactive (or both), as discussed above. Indeed, methods, including those developed by 10X Genomics (26) and BD Biosciences (27), allow for the identification of single cell, paired TCR or BCR sequencing, generating data on true clonality. Thus, TCR and BCR sequencing in conjunction with scRNAseq will provide invaluable insight into the clonal immune response in organ rejection and gives a potential pathway toward alloantigen identification.

### Additional Approaches Coupled to scRNAseq

While scRNAseq can determine the transcriptional landscape of individual cells, it does not provide information regarding the epigenetic background of those cells. Recent developments have enabled a single-cell assay for transposase accessible chromatin (scATACseq) (28), which identifies regions of open chromatin by virtue of transposase insertion at such regions. Thus, combining scRNAseq with scATACseq is a very powerful approach to understanding potential gene regulatory networks (GRNs) as well as cell lineage analysis (120–123). In addition to performing scATACseq, bioinformatic approaches also are instrumental in identifying GRNs. Techniques such as SCENIC (124) have been developed to map out GRNs, which can be used to determine cellular states and cluster cells based on regulatory subnetwork activity. This computational approach can also be used to reconstruct the cellular differentiation trajectory. In solid organ transplant rejection, this epigenetic and genetic data could help identify the gene regulatory networks utilized by alloreactive T cells that enable their developmental trajectory, proliferative expansion, and functional differentiation. Deeper mechanistic understanding to these GRNs will undoubtedly lead to novel therapeutic targets to mitigate allograft rejection.

Despite the powerful information that can be obtained from mRNA, gene expression analyses do not necessarily translate into protein expression. Integrating scRNAseq data with mass cytometry or cytometry by time of flight (cyTOF) allows for simultaneous analysis of mRNA and protein (29, 30). Additional techniques, including cellular indexing of transcriptomes and epitopes by sequencing (CITEseq) (31) and RNA expression and protein sequencing (REAPseq) (32) also allow for a combined measurement of mRNA and protein expression of cells. Index sorting also helps link protein and gene expression from single cells together by first using fluorescently labeled antibodies to sort cells into wells of a plate before RNA sequencing each well (33). However, limitations of index sorting include not only the flow rate of the sorter, but also the number of cells assayed, which is limited by the number of wells in the plate. This leads to a much lower throughput from index sorting as compared to other methods (**Table 1**). Nevertheless, these approaches in measuring both mRNA and protein expression on the same cells validate findings obtained and facilitate the understanding of pathways and proteins of interest. Thus, the ability to combine genomic and protein expression analyses in conjunction with scRNAseq make it a highly beneficial tool in understanding the cellular landscape during organ rejection.

Since the transcriptomic profile of cells in the allograft are minimally understood after immunosuppressive therapies, scRNAseq techniques can be leveraged to understand changes in the cellular composition of the allograft after various treatments. With sequential allograft biopsies, scRNAseq can help delineate not only the transcriptomic profiles of cells in refractory rejection, but also how these cell interactions change after various anti-rejection therapies. This allows for the interrogation and hypothesis generation of alternative pathways that could be responsible for immunosuppressive escape and persistent rejection, and potentially identify new therapeutic targets. The development of multiple computational software analysis programs provides the opportunity to dive even deeper into cells and pathways essential in immunosuppressive escape and persistent allograft rejection. By generating data on the gene expression of individual cells, receptor-ligand pairs can help determine cellular interactions. Software programs such as CellChat (125) aid in understanding intercellular communications by using pattern recognition approaches to predict signal coordination. Another program, RNA Velocity (126), uses an estimated time derivative to predict the future of individual cells on a timeline. In a mouse model of kidney transplant, this technique was used to follow the path of differentiation of intragraft myeloid and helped to identify a gene, *Axl*, that they showed plays a role in intragraft myeloid and T cell differentiation (127). Programs such as RNA Velocity can be leveraged to not only understand the lineage tree of cells of interest in a rejecting allograft, but also possibly predict response to therapy through determination of the future gene expression of these cells.

Despite all the benefits scRNAseq could provide in the field of transplantation, caveats of this technique have made mass analysis of allograft acceptance and rejection difficult. Depending on the approach used, only relatively few numbers of cells can be assessed. Further, even though receptor-ligand pair interactions can help infer cell to cell communication, these interactions cannot be confirmed due to lack of spatial context. In addition to the aforementioned challenge with tissue dissociation techniques and enzyme activity temperatures, taking cells, especially endothelial cells, out of their native context leads to rapid death and dissemination of cellular mRNA, leading to potential contamination and increased number of mitochondrial reads. Due to the challenge of analyzing cellular interactions and using techniques that involve tissue dissociation, there has been a large interest in utilizing scRNAseq techniques in a spatially resolved manner, leading to the development of spatial transcriptomics.

## Spatial Transcriptomics

While scRNAseq gives tremendous data on individual cells, it lacks histologic contextual information. The next iteration of transcriptomic analysis is that of spatial transcriptomics (ST), which analyzes transcriptomic data on histological tissue sections. A substantial advantage of this ST approach is that the locations of cells of interest can be determined, and cellular interactions can be interrogated in their native context. This provides an advantage over scRNAseq, as the requirement for tissue dissociation into a single cell suspension in scRNAseq limits understanding of cellular communications (**Table 1**).

A vast majority of techniques used for spatial transcriptomics involve hybridization-based assays. Methods using probe hybridization utilize techniques developed for *in situ* hybridization but include capture of a substantial number of probes instead of one. Several spatial platforms have been developed, including seqFISH (34, 128), smFISH (129, 130), and MERFISH (35, 131). seqFISH utilizes multiple rounds of barcoding hybridization using probes with a fluorescence marker attached. After each round of hybridization, the tissue is imaged, and the probes are washed off. By using consecutive rounds of hybridization and washing, a greater number of transcripts can be investigated. Unique transcripts can be identified by using a combination of signals from each round of barcoding. However, due to multiple rounds of hybridization of washing, this technique is time consuming. Additionally, selection of the probes for hybridization requires *a priori* knowledge of the genes of interest. smFISH and MERFISH use similar techniques, where smFISH uses spectrally resolved probes and MERFISH uses error-corrected barcodes. By using error-corrected barcodes, MERFISH aims to reduce the amount of false positive or false negative barcodes that can be generated through multiple labeling rounds, giving a crisper picture of the transcriptomic profile of the tissue. Another advantage of MERFISH is that due to its single cell resolution, it enables the incorporation of TCR and BCR profiling, which allows for the determination of the relative location of expanded clones. Similar to scRNAseq, this gives clues to cellular clonality and potential specificity. However, one major concern of these techniques is spectral overlapping through density saturation. seqFISH+ (34) overcomes this density concern, and data obtained from seqFISH+ has been shown to cluster similarly to scRNAseq data on the same samples.

Another technique is NanoString’s GeoMx Digital Spatial Profiler (36, 37), which uses oligo-labeled antibodies or probes with a UV photocleavable linker. A unique aspect of this system is that the machine includes an autoconfiguring mirror that allows for separation of specific components of the tissue, such as separating the immune cells from tissue epithelial cells. This allows for in-depth analyses of individual components of the tissue, providing a clearer tissue image and transcriptomic outline. However, this technique shares a similar limitation as those mentioned above – there is a lack of transcriptome-wide analysis due to the need of prior knowledge of the probes or antibodies of interest. 10X Genomics’ Visium overcomes this issue by using a transcriptomic-wide approach to interrogate tissue sections. To do this, Visium requires tissue sections placed on special microscope slides that have barcoded reverse transcriptase primers, allowing for the mRNA of the tissue to hybridize once permeabilized. These primers also enable the identification of the location of the transcripts once the cDNA is extracted for library preparation and sequencing. Currently, the barcoded regions do not allow spatial resolution down to a single cell level (currently 5-10 cells), which limits the understanding of regulatory networks and cellular interactions. However, there are plans to achieve a single cell resolution in the near future, making this a valuable approach for unbiased, whole cell transcriptomic analysis in a spatially resolved manner. Furthermore, both the technologies from Nanostring and 10X have been developed to accommodate both fresh and preserved tissues. The ability to use ST techniques on FFPE tissue is a huge breakthrough in using this technique as it enables the analysis of banked tissue, especially since FFPE tissue is how Banff scores are primarily obtained. However, a challenge of using banked FFPE tissue is the potential for RNA to be chemically modified and degrade over time, reducing the sensitivity of this approach.

In additional to probe-based approaches for spatially resolved transcriptomics of mRNA expression, other techniques allow for the analysis of protein expression on histological tissue sections. These techniques include multiplexed spatial protein analytical methods such as co-detection by indexing (CODEX^®^) technology or mass cytometry imaging. Both these techniques utilize conjugated antibodies to tag the histological tissue section prior to imaging. Similar to the probe-based approaches discussed, CODEX^®^ leverages repeated fluorochrome conjugation and removal to increase the number of markers that can be assessed (38). Additionally, similar to CITEseq, CODEX^®^ enables in-depth analysis of clustering based on particular antibodies of interest. In contrast, Fluidigm’s Imaging Mass Cytometry^™^ parallels CyTOF by using metal ion-tagged antibodies instead of fluorochromes. This mitigates concerns of antibody staining order or serial staining and marker removal (132). Although it is currently not possible to perform RNA probe-based and protein-based techniques on the same tissue section simultaneously, performing these techniques on separate tissue sections from the same histological block could provide invaluable information on mRNA and protein expression.

Due to their novelty, there has been little use of ST to investigate solid organ transplant rejection, but given the preliminary use of these techniques in other fields such as cancer research (133–135) and developmental biology (136), ST will be an invaluable tool in the analysis of organ rejection, providing understanding of not only the transcriptomic profile of rejection, but also overlaying that on the histologic of the rejecting allograft. Additionally, because ST techniques also allow for either hematoxylin and eosin (H&E) or immunofluorescence staining prior to tissue permeabilization, determination of Banff criteria on the same biopsy core and tissue slice as the transcriptomic analysis are possible. This would ensure the transcriptomic data match the pathology report. Thus, integration of spatial transcriptomic approaches to understand allograft rejection will be revolutionary.

## Conclusions

Technologies used to understand solid organ allograft rejection have come a long way in the past 15 years, from the use of microarrays to understand genes relevant in TCMR and AMR to new avenues to interrogate rejection with single cell resolution (e.g., scRNAseq, scATACseq, spatially resolved transcriptomics). The success of microarray technology, as seen with the molecular microscope diagnostic technique, has paved the path for newer technologies to potentially make a similar transition from bench to bedside. However, current techniques of scRNAseq or spatial transcriptomics are not streamlined enough for clinical diagnostics. Nevertheless, with the speed of advancing new technologies, it is possible these techniques will be standard in the near future. In addition to the aforementioned techniques that allow interrogation of the transcriptome of rejecting allografts, donor-derived cell-free DNA (ddcfDNA) has also shown potential to be a valuable tool in the clinical analysis of transplant rejection. The ability for ddcfDNA to measure allograft damage directly and quantifiably, coupled with the non-invasive nature of the peripheral blood draw used to obtain the sample, makes the use ddcfDNA in transplantation an attractive alternative to biopsies. Even though peripheral blood sampling may not properly recapitulate cellular mechanisms of rejection in the allograft, the utility of assessing for biomarkers should not be underestimated. However, sampling of the urine or BAL may be a better non-invasive test for predicting allograft rejection in the kidney or lung, respectively. Nevertheless, widespread implementation of scRNAseq technology to understand allograft rejection and cellular response to therapy will play an instrumental role in furthering the understanding of solid organ transplantation, paving the way for newer, targeted, and personalized therapeutics.

## Author Contributions

TS was responsible for preparation of the manuscript. KR, BB, DH, and EW were responsible for manuscript revisions and editorial input. All authors contributed to the article and approved the submitted version.

## Funding

This work was supported by the National Institutes of Health (R21AI142264 [DH, EW], R01AI154932 [DH, EW], R35GM118166 [BB]) and by Novartis Pharmaceuticals. Novartis Pharmaceuticals had no involvement in the preparation of this review manuscript.

## Conflict of Interest

The authors declare that the research was conducted in the absence of any commercial or financial relationships that could be construed as a potential conflict of interest.​​​

## Publisher’s Note

All claims expressed in this article are solely those of the authors and do not necessarily represent those of their affiliated organizations, or those of the publisher, the editors and the reviewers. Any product that may be evaluated in this article, or claim that may be made by its manufacturer, is not guaranteed or endorsed by the publisher.
